# Evaluation of Preventive Treatment Protocols for Patients under Antiresorptive Therapy Undergoing Tooth Extraction at a Swiss University Clinic

**DOI:** 10.3390/ijerph18189924

**Published:** 2021-09-21

**Authors:** Ellen Pick, Nicolas Leuenberger, Irina Kuster, Nicole Selina Stutzmann, Bernd Stadlinger, Silvio Valdec

**Affiliations:** 1 Center of Dental Medicine, Clinic of Cranio-Maxillofacial and Oral Surgery, University of Zurich, 8032 Zurich, Switzerland; ellen.pick@zzm.uzh.ch (E.P.); nicolas.leuenberger@zzm.uzh.ch (N.L.); irina.kuster@zzm.uzh.ch (I.K.); bernd.stadlinger@zzm.uzh.ch (B.S.); 2 Statistical Services, Center of Dental Medicine, University of Zurich, 8032 Zurich, Switzerland; nicole.stutzmann@zzm.uzh.ch; 3 Department of Stomatology, Division of Periodontology, Dental School, University of São Paulo, Butantã 2227, SP, Brazil

**Keywords:** ARONJ, MRONJ, Bisphosphonates, Denosumab, osteoporosis, osseous metastasis, risk profiles, preventive protocols, antibiotic prophylaxis, primary wound closure

## Abstract

Antiresorptive agent-related osteonecrosis of the jaw (ARONJ) is a dreaded complication in patients with compromised bone metabolism. The purpose of the present study was to examine the occurrence of ARONJ and its related factors among patients with a history of antiresorptive therapy undergoing tooth extraction using preventive protocols at a Swiss university clinic. Data were retrospectively pooled from health records of patients having received a surgical tooth extraction between January 2015 and April 2020 in the Clinic of Cranio-Maxillofacial and Oral surgery, University of Zurich. A total of 970 patients received an extraction with flap elevation or wound closure during this period. A total of 104 patients could be included in the study. Furthermore, variables including age, gender, smoking, risk profile, choice, indication and duration of antiresorptive therapy, number of extractions, extraction site, surgical technique, choice and duration of antibiotics as well as the presence of postoperative inflammatory complications were assessed. Overall, 4 patients developed ARONJ (incidence of 3.8%) after tooth extraction at the same location, without previous signs of osteonecrosis. Preventive methods included predominantly primary wound closure using a full thickness mucoperiosteal flap and prolonged perioperative antibiotic prophylaxis. In accordance with current literature, the applied protocol showed a reliable outcome in preventing ARONJ when a tooth extraction is required.

## 1. Introduction

Antiresorptive agent-related osteonecrosis of the jaw (ARONJ) is a serious and impairing complication [1]. It affects patients undergoing Bisphosphonate or Denosumab therapy for a variety of bone diseases such as osteoporosis and bone metastasis [2,3].

In Switzerland, around 20% of women and 7% of men over the age of 50 suffer from osteoporosis; for them, antiresorptive agents still remain the first choice of treatment [4,5]. With the aging of the general population, it is safe to assume that the prescription of antiresorptive medications will continue to increase. Therefore, the dental practitioner will be more frequently confronted with patients at risk of osteonecrosis of the jaw [1,6,7]. In addition, osteoradionecrosis (ORN) represents another challenge for dentists. Although showing the same clinical presentation as ARONJ, physiopathological characteristics differ [8]. Nevertheless, both osteonecrosis forms represent an important public health issue, particularly in the oncologic field, where current research focuses on decreasing the incidence of these debilitating pathologies [9].

Tooth extraction has previously been determined as the primary cause of the development of ARONJ [10]. However, recent studies have reported that the pre-existing periodontal infection of the tooth rather than its surgical removal is the initiating factor that triggers osteonecrosis [3,11,12]. Therefore, the extraction of an unrestorable, infected tooth in patients undergoing antiresorptive therapy might result in a reduction of the risk of developing ARONJ, when appropriate preventive measures are applied [3].

In recent years, a considerable amount of prophylactic treatment protocols for tooth extraction in patients at risk of ARONJ have been introduced [1,6,13,14,15]. However, data evaluating the clinical outcome in patients being treated with these preventive guidelines remain sparse.

The aim of this study was therefore to evaluate the preventive treatment protocol used in the university clinic of Zurich, for tooth extraction in patients under antiresorptive therapy by assessing the incidence of ARONJ. Furthermore, a variety of parameters including age, gender, smoking, risk profile, choice, indication and duration of antiresorptive medication, number of extractions, extraction site, surgical technique, choice and duration of antibiotics as well as the presence of postoperative inflammatory complications were evaluated.

## 2. Materials and Methods

The present study was conducted at the Clinic of Craniomaxillofacial and Oral Surgery at the Center of Dental Medicine, University of Zurich, and approved by the ethical committee of the canton of Zurich (BASEC-Nr. 2020-01120).

Data were collected retrospectively by screening the health records of patients who received a single or multiple tooth extraction with primary wound closure between January 2015 and April 2020. Inclusion criteria were: male or female, over 18 years old, under current or previous antiresorptive therapy (Bisphosphonates or Denosumab), tooth extraction with primary wound closure. The exclusion criteria were defined as the follows: (1) patients who received a tooth extraction with primary wound closure without a history of antiresorptive therapy; (2) patients who did not provide a written consent for the use of their medical data for academic purposes; (3) patients under the age of 18 and (4) patients with a history of head and neck radiotherapy.

In our clinical practice, tooth extractions in patients under antiresorptive therapy were performed following the German AWMF guidelines [16], which include the following preventive measures: (1) prolonged peri-operative antibiotic prophylaxis, starting at least 24 h before the intervention, using Amoxicillin, Amoxicillin/Clavulanic acid or Clindamycin if penicillin allergy was present, (2) use of an atraumatic extraction technique, (3) smoothening of sharp bony edges after extraction, (4) primary wound closure with tension-free sutures, (5) the use of liquid and/or soft foods and (5) regular postoperative follow-ups until complete mucosal wound healing. The patients in our cohort were additionally given a mouth rinse containing chlorhexidine starting the day of the intervention until 7 days postoperatively. Furthermore, one follow-up was performed 1 week postoperatively, and sutures were removed after 2 weeks during the second follow-up, when complete mucosal healing was achieved. Within the anamnestic protocol, in collaboration with the antiresorptive drug prescriber, a potential drug holiday was considered for each patient.

After primary evaluation, the data of the included patients were encrypted, and the occurrence of ARONJ was assessed for each case. In this study, ARONJ diagnosis was based on the AAOMS definition: patients under current or previous antiresorptive therapy presenting with exposed bone or bone that can be probed through an intraoral or extraoral fistula in the maxillofacial region that has persisted for longer than 8 weeks in absence of a history of radiation therapy to the jaws or obvious metastasis disease to the jaws [2].

The incidence rate of ARONJ was calculated including patients who presented with osteonecrosis at the site of previous tooth extraction in absence of previous signs of ARONJ and was described for the number of patients and number of sites. Regarding the antiresorptive medication, variables such as indication of prescription (osteoporosis, multiple myeloma, osseous metastasis); type of medication (Bisphosphonate or Denosumab); duration and route of administration (intravenous, peroral or subcutaneous) were investigated. Based on these parameters, patients were classified in 3 different risk profiles (low, medium and high) according to the AWMF guidelines [16] (see Table 1). Variables related to the surgical intervention included extraction site (front tooth, premolar or molar); extraction number; surgical technique for achieving primary wound closure; type and duration of antibiotic prophylaxis; postoperative occurrence of inflammatory complications (wound dehiscence, pain, swelling, redness); and the need for a surgical reintervention. Finally, patient-related characteristics such as age, gender and smoking were assessed.

The statistical analysis was performed using the statistical software R, version 4.0.2 (R Foundation for Statistical Computing, Vienna, Austria) [17], including the package tidyverse [18]. For each variable evaluated, the mean and percentage value were assessed. For parameters including age, the duration of antiresorptive therapy and duration of antibiotic therapy, the standard deviation was added.

## 3. Results

**Figure 1:**
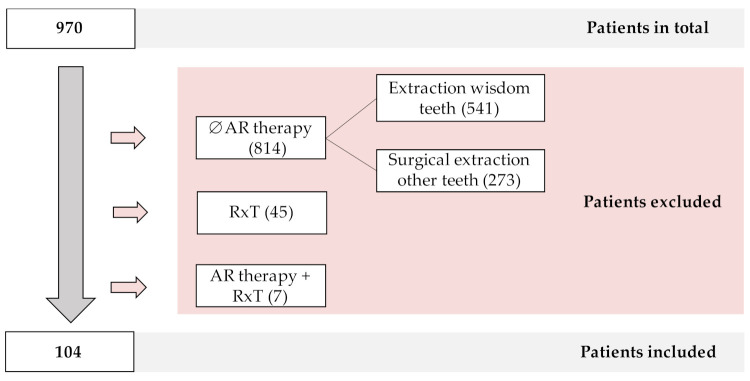
Application of exclusion criteria to the total number of patients evaluated. AR = antiresorptive therapy, RxT = radiotherapy.

Overall, 970 patients received a tooth extraction with simultaneous flap raising or primary wound closure between January 2015 and April 2020. In most cases, this procedure was performed in patients without antiresorptive medication (routine removal of wisdom teeth (541) and other teeth (273)). As a preventive measure, patients with a history of maxillofacial radiotherapy received primary wound closure following tooth extraction (45). These two groups with a total of 859 patients were excluded from the study. Further, seven patients having current or previous antiresorptive therapy during also had a history of maxillofacial radiotherapy and were excluded. A total of 104 patients met all the inclusion criteria (see Figure 1).

### 3.1. Characteristics of Study Population and Antiresorptive Medication

Regarding the characteristics of the patient-cohort investigated (summarized in Table 2), the mean age of the patients was 71.54 ± 12.04 years. A higher percentage of females (75%) compared to males (25%) could be observed. Referring to smoking, more patients were non-smokers (*n* = 77) compared to smokers (*n* = 27). The most frequent indications for the prescription of the antiresorptive therapy was osteoporosis (*n* = 67), followed by osseous metastasis (*n* = 14) and multiple myeloma (*n* = 5). Therefore, due to the osteoporotic medication scheme, most of the patients (*n* = 66) had a low risk profile. The medium risk category included 18 patients, while 19 presented a high risk of developing ARONJ. The antiresorptive agent of choice for osteoporosis was Prolia^®^ (Denosumab) given subcutaneously (*n* = 38), followed by Ibandronat (*n* = 28) given intravenously (82.14%), whereas for malignant indications Xgeva^®^ was the most common. The mean duration of antiresorptive therapy at the time of the extraction was 4.08 ± 3.30) years.

### 3.2. Extraction Number and Site, Surgical Technique, Antibiotic Prophylaxis and Postoperative Inflammatory Complications

**Figure 2:**
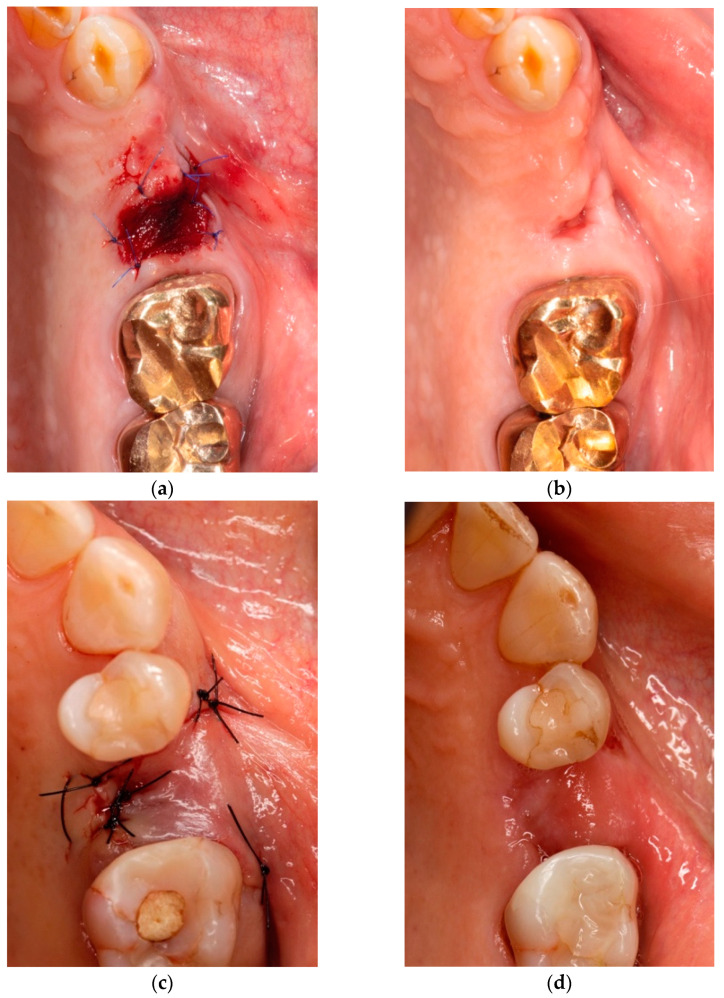
(**a**) Patient 1: primary wound closure after extraction of tooth 25 with a xenogenic collagen graft (mucograft seal^®^); (**b**) postoperative follow-up 3 weeks after placing the graft in patient 1; (**c**) Patient 2: primary wound closure after extraction of tooth 25 using a full thickness mucoperiosteal flap; (**d**) postoperative follow-up 4 weeks after plastic wound closure in patients 2.

Overall, 203 tooth extractions were performed. Molars (*n* = 95) and premolars (*n* = 65) were more frequently removed than frontal teeth (*n* = 43). Teeth in the lower jaw (*n* = 108) were slightly more often extracted compared to teeth in the upper jaw (*n* = 95). Most extractions were performed whilst the patients were under current antiresorptive therapy (74.38%). For most cases, the tooth extraction was followed by a primary wound closure using a full thickness mucoperiosteal flap (93.59%). A collagen graft (mucograft seal^®^) was used in four extraction sites in three patients with a low and one patient with a medium risk profile (see Figure 2). Antibiotic prophylaxis was prescribed on a regular basis. Prophylactic antibiotic medication of choice was Amoxicillin (64.03%), followed by Co-amoxicillin (17.24%) and Clindamycin (13.30%) with an overall duration of administration of 2.20 ± 0.79) weeks. Wound dehiscence appeared in 14 extraction sites (23.33%). After surgical revision, having been performed in most cases, five sites developed ARONJ (55.55%). Postoperative inflammation was present in 60 extraction sites (29.55%), with pain (45%) and hematoma (35%) being the most frequently observed (see Table 2).

### 3.3. Occurrence of ARONJ

**Figure 3:**
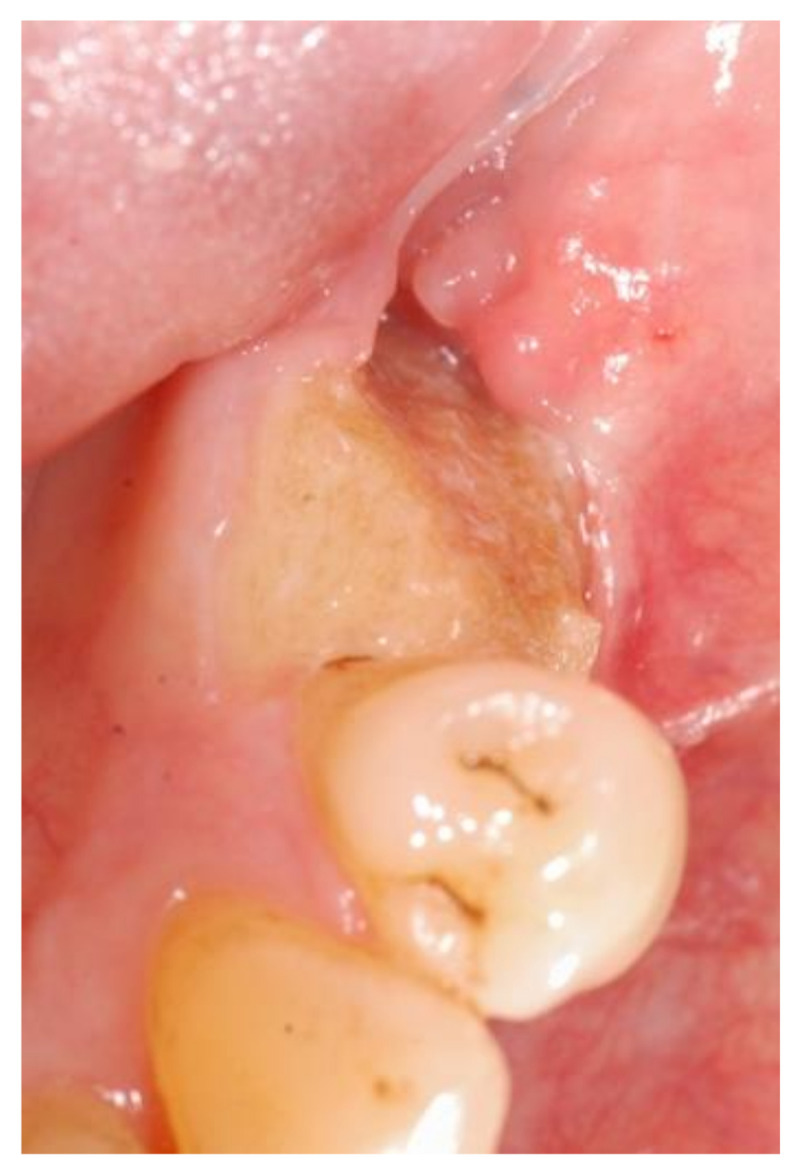
Example: a 69-year-old male patient from our cohort, presenting with ARONJ after extraction of tooth 35. This patient was undergoing Xgeva^®^ therapy for 4 years due to a prostate carcinoma and therefore presented a high risk profile.

Out of 104 patients (*n* = 203 extraction sites), ARONJ was diagnosed in four patients (*n* = 4 extraction sites), in absence of previous clinical signs of osteonecrosis. An incidence rate of 3.8% (1.9% of extraction sites) was therefore obtained. Overall 21 patients (29 extraction sites) developped ARONJ in our patient-cohort (see Figure 3.). The majority of the sites (*n* = 22) already presented with a preexisting osteonecrosis prior to tooth extraction. In these cases, a surgical revision accompanied by the extraction of another tooth in the same area as the already affected bone was performed. Four extraction sites showed a persisting ARONJ post revision (before and after). Three patients developed ARONJ at the same site and one patient both the same and at a different site than the performed tooth extraction. In addition, 15 extraction sites in total developed ARONJ at another localization unrelated to the tooth extraction (see Table 3).

### 3.4. Characteristics of Patients Presenting with ARONJ

#### 3.4.1. Total ARONJ Patients

The overall patients who developed ARONJ had a low risk profile in 12, medium in 3 and high risk in 6 cases (see Table 4). Women (*n* = 17) were more frequently affected than men (*n* = 4) and most of them were non-smokers (66.67%). ARONJ developed mostly after extraction of premolars (*n* = 9) in the lower jaw (*n* = 18). Moreover, dehiscence and pain were the predominant inflammatory complications present. Referring to the medication, most ARONJ cases were treated with Prolia^®^ (Denosumab) subcutaneously (33.33%), most of the patients were under current antiresorptive therapy (65.52%) and were taking the antiresorptive agent for a mean of 4.85 ± 4.93) years at the time of extraction.

#### 3.4.2. Patient with ARONJ after Extraction, at the Same Location, without Previous Signs of ARONJ

The four patients who developed ARONJ had a high risk profile in half of the cases (see Table 4). Females and males were equally affected and more smokers (75%) than non-smokers (25%) were observed. The extraction of molars (*n* = 3) in the upper jaw (*n* = 3) were the most frequently associated with ARONJ in these patients. Postoperatively, all the patients presented with inflammatory complications, with dehiscence being the most frequent, followed by pain and redness. Regarding antiresorptive medication, Xgeva^®^ (Denosumab) was the most often administered (50%), all the patients were under current antiresorptive therapy during the intervention and the mean duration of the therapy was 2.00 ± 0.81) years at the time of extraction. None of the patients received a drug holiday prior to tooth extraction.

## 4. Discussion

This study analyzed the development of ARONJ after tooth extraction in patients with a history of antiresorptive drug intake. By applying perioperative antibiotic prophylaxis and primary wound closure, an overall incidence of ARONJ of 3.8% was obtained. This is comparable to the incidence rate (2.4%) reported by Spanou et al. [7], suggesting that the above-mentioned preventive measures showed a reliable outcome. Indeed, according to a recent Italian consensus update from 2020, antibiotic prophylaxis and primary wound closure still remain the two pillars of the preventive protocol for patients requiring a tooth extraction with a history of antiresorptive therapy [19]. Although the effective ARONJ incidence after tooth extraction was low, a considerable number of patients showed clinical signs of osteonecrosis prior to tooth extraction at the same site. Most of these cases presented a complete mucosal healing after surgical intervention, which is in line with the findings of Otto et al., suggesting that these preventive measures can also be successfully applied for the management of ARONJ [3].

In our patient cohort, primary wound closure was mostly achieved using a full thickness mucoperiosteal flap. This surgical procedure implicates the denudation and incision of the periosteum to enable flap mobilization, which is accompanied by a reduced blood supply of the underlying bone [16]. Furthermore, due to the coronal relocation of the muco-gingival margin and the reduced height of the vestibulum, prosthetic rehabilitation with dental implants or removable prosthesis can be hindered. This may reduce the quality of life of these patients [20].

Current literature shows a new tendency of using less invasive surgical protocols for patients with a low risk of ARONJ to avoid unnecessary overtreatment [3]. The closure of the post-extraction socket with a xenogenic collagen graft (mucograft seal^®^) is a preventive concept that has been introduced for this indication [20]. The application of platelet-rich fibrin (PRF) in the post-extraction socket as a healing promoter has also shown promising results [15]. Finally, Pippi et al. even suggested that healing of the extraction socket by secondary intention is a viable treatment option in patients presenting a low risk profile, as long as it concerns non-surgical tooth removal [21].

For ARONJ prevention, most authors agree that a perioperative antibiotic prophylaxis should be given when performing a tooth extraction [3,7,13,19,22,23,24]. In this case, the antibiotic administration starts prior to surgery and is prolonged after the intervention, usually until mucosal healing is completed [13,25]. However, there is great variation regarding recommendations for the choice, dosage schedule and particularly the duration of antibiotic prophylaxis in the literature [13,26]. Geographical localization seems to play a significant role in this heterogeneity. For instance, Clindamycin remains one of the most frequently prescribed antibiotic agents in Germany and is part of the recommended list of antibiotics in the German AWMF guidelines, whereas in other countries, other antibiotic agents are more commonly used [13,16,27]. In addition, the co-administration of different antibiotic agents has been proposed for ARONJ prevention [13]. For example, the authors of the Italian consensus update 2020 suggest using a combination of Amoxicillin/Clavulanic acid and Metronidazole to achieve a higher-spectrum perioperative antibiotic regimen [19].

Despite a lack of consensus, a general trend of reducing the duration of antibiotic administration to prevent bacterial resistance has been increasingly discussed in the last years. Prolonged antibiotic therapy has not only been linked to a facilitated selection of resistant bacterial strains, but can also cause delayed allergic reactions and considerable gastro-intestinal side effects [23,28,29,30]. This rising public health issue should therefore be considered whilst prescribing antibiotic prophylaxis. The duration of the therapy should be adapted to each patient according to their ARONJ risk profile. In our clinical practice, antibiotic coverage is usually started 24 h before surgery for patients with a low and medium risk profile, whereas for patients with a high risk profile, antibiotic administration is initiated 48 h before the intervention. In our patient cohort, the antibiotic regimen was most frequently pursued for 2–3 weeks postoperatively. Especially for patients with a low risk profile, this may be reduced in the future.

Another factor influencing the general lack of consensus regarding guidelines for ARONJ prevention is the heterogenous definitions of the different international risk categories. The American Association of Oral and Maxillofacial surgeons (AAOMS) position paper suggested that regardless of the type of antiresorptive medication, cancer patients had a much higher overall risk of ARONJ compared to osteoporosis patients. In addition, longer time period of antiresorptive therapy showed a higher risk to develop ARONJ. Furthermore, potential co-factors such as antiangiogenetic medication, corticosteroid therapy, anemia and diabetes have been associated with a higher risk of osteonecrosis. Although, most guidelines generally agree with these findings, unanimously accepted risk categories do not exist in current literature. The German AWMF guidelines describe 3 different risk profiles, whilst the Italian consensus presents a different method of categorizing ARONJ risk, focusing on the duration of the antiresorptive therapy [2,16,19].

In our study, most patients who developed ARONJ presented a low risk profile. This is most likely caused by the fact that overall, antiresorptive medication was more frequently administered for the treatment of osteoporosis than for malignant diseases in this cohort. Therefore, patients with a medium and high-risk profile still proportionately presented more frequently with ARONJ. This underlines the importance of adapting preventive treatment protocols to a specific and clear-defined patient risk category. As mentioned before, ARONJ prevention methods are starting to move towards minimally invasive procedures, which have only been introduced to patients with a low risk of ARONJ. For patients presenting a higher risk of osteonecrosis, tooth extraction followed by strict primary wound closure and prolonged perioperative antibiotic prophylaxis remains mandatory.

In the present study, both patients with previous and current antiresorptive therapy at the time of tooth extraction were included. ARONJ patients were most frequently under ongoing antiresorptive therapy whilst the intervention was performed. This suggests that the ARONJ incidence was lower when the antiresorptive drug had been discontinued. Therefore, a drug holiday prior to tooth extraction represents another ARONJ prevention concept worth mentioning [2,19,31]. Depending on the type of patients (cancer versus non-cancer patients), the duration and type of medication (Bisphosphonates versus Denosumab), a balance between the ARONJ risk and the benefit of the antiresorptive drug can be assessed [19]. With a more recent introduction of Denosumab, which compared to Bisphosphonates, has a shorter half-life, and does not accumulate in the bone, some authors have suggested that a drug holiday might reduce the ARONJ risk [32,33]. However, for cancer patients taking high doses of Denosumab, the risk of skeletal-related events (SRE) or progression of metastasis is often greater than the ARONJ risk itself. In contrast, after 2 years of antiresorptive treatment, the ARONJ risk potentially starts overweighing the risk of morbidity due to skeletal complications or metastasis progression [19]. Due to the current lack of universal agreement and high-evidence data regarding the effectiveness of a drug holiday in reducing ARONJ incidence, the antiresorptive drug prescriber and dental practitioner should work closely together to evaluate each patient’s individual risk-benefit balance [19,31,32]. After thorough consideration in collaboration with the general physician, a drug holiday was applied when deemed appropriate in our patients. We believe the drug holiday to be an important aspect of ARONJ prophylaxis, in accordance to recently published guidelines [19].

The assessment of the quality of life (QoL) of patients taking antiresorptive medication represents another recent concept of interest in ARONJ prevention. A systematic review conducted by Bensi et al. showed that the QoL was significantly reduced both in cancer and non-cancer patients affected with ARONJ, with pain being the most important influencing factor. It was therefore suggested that conducting a thorough ARONJ prevention protocol should include regular monitoring of the QoL of patients under antiresorptive therapy [34].

Despite the proven effectiveness of current preventive protocols for tooth extraction in patients under antiresorptive agents, a significant reduction of the risk of ARONJ in the general population can only be achieved if routine dental focus-screenings and treatment precede the initiation of the antiresorptive therapy [2,19,35]. Therefore, a sensibilization and training-guide for nurses and allied healthcare professionals to promote the use of these dental screenings has recently been introduced [36]. Targeted questionnaires for ARONJ-related drug prescribers have also been established to further enhance consciousness. Although the above-mentioned caregivers find themselves at the forefront of the primary prevention, awareness should also be extended to dentists and dental hygienists. Bruckmoser et al. showed that knowledge regarding ARONJ prevention in dentists from German-speaking countries was insufficient and therefore recommended that especially oncology patients, at higher risk of ARONJ, should be referred to oral surgeons or specialized institutions where dental focus-screenings are regularly performed [37]. Considering the frequent prescription of antiresorptive agents in the Swiss population, general dental practitioners are routinely confronted with patients under antiresorptive drugs. The identification of patients presenting a higher risk of ARONJ and referring them to a specialized surgery-based facility is therefore of utmost importance. Raising awareness about primary and secondary ARONJ prophylaxis should begin with the education of dental students in university-settings to encourage a close-knitted collaboration between future dental practitioners in private practice with specialized oral surgeons and antiresorptive drug prescribers.

Regarding the limitations of the study, the heterogeneous pre-treatment history of the included patients, having been referred to our clinic from different dental institutions or medical facilities, as well as the retrospective study design, only allowed for a descriptive statistical analysis. Since ARONJ is a rare condition [38], cohorts including a higher number of patients are necessary to determine the real significance of patient-, medication- and intervention-related parameters as potential risk factors for the development of ARONJ. Prospective, controlled studies evaluating the above-mentioned variables are needed to assess their role in the onset of ARONJ in oral medicine. In Switzerland, there is a growing tendency among doctors prescribing focus-screenings before initiating antiresorptive therapy [39]. However, the costs of this examination is not always covered by health insurance [40]. Revising the current health care policy to create accessibility to dental focus-screenings for all patients prior to antiresorptive therapy initiation might allow to create a baseline oral health status. Therefore, more homogenous patient-cohorts could be evaluated in future studies. Another limitation of the study is the lack of histopathological analysis of the bone quality. Bone biopsy at the time extraction is favorable to determine a potential pre-existing osteonecrosis of the jaw.

## 5. Conclusions

According to previous literature and the current findings of this study, ARONJ can be efficiently prevented using perioperative antibiotic prophylaxis and primary wound closure when tooth extraction is necessary. Nevertheless, dental focus-screening and treatment in patients prior to initiating antiresorptive therapy should be mandatory for ARONJ prophylaxis. Care should be taken to appropriately teach dental students and raise awareness among dental and medical practitioners in private practice about primary prevention and management of patients taking antiresorptive medication. Finally, incorporation of new preventive treatment modalities should be further encouraged an investigated especially for patients with a low risk of developing ARONJ.

## Figures and Tables

**Table 1:** Risk profiles according to the AWMF guidelines, with associated ARONJ risk in % determined by the indication, type of medication, route of administration, frequency/dose of the antiresorptive agent [16].

	Low-Risk 0–0.5%		Medium-Risk 1%		High-Risk 1–21%	
Indication	Primary osteoporosis		Therapy-induced Osteoporosis Prevention of SRE ^2^ Co-medication with immunomodulators ^3^ Additional risk factors ^4^		Osseous metastasis Multiple myeloma	
Medication/Route of administration	BP (p.o, i.v) ^1^	DNO (s.c) ^1^	BP (i.v)	BP (i.v) + immunomodulators	BP (i.v)	DNO (s.c)
Examples of dosage and frequency of administration every *n* month (M) or week (W)	Zoledronat 5 mg/12 M Ibandronat 3 mg/3 mL/3 M	60 mg /6 M	Zoledronat 4 mg/6 M		Zoledronat 4 mg/4 W	120 mg/4 W

^1^ BP = Bisphosphonates, DNM = Denosumab, p.o = peroral, i.v = intravenous, s.c = subcutaneous; ^2^ SRE = skeletal-related events in cancer patients; ^3^ For example: methotrexate for the treatment of rheumatoid arthritis; ^4^ Additional systemic factors influencing wound healing including anemia, diabetes, hyperparathyroidism, dialysis, chemotherapy, glucocorticoid therapy, treatment with angiogenesis inhibitors and advanced age.

**Table 2:** Characteristics of study population.

	Variables Related to Number of Patients		
Parameter	Category	Result	Percentage (%)
Age	Years (mean ± SD)	71.54 ± 12.04	
Gender	Male	26	25.00
	Female	78	75.00
Smoking	Yes	27	25.96
	No	77	74.04
Risk profile	Low	66	63.46
	Medium	18	17.30
	High	19	18.26
	No information	1	0.96
Antiresorptive drug/-- Route of administration	Prolia^®^ (subcutaneous)	38	36.53
	Xgeva^®^ (subcutaneous)	9	8.65
	Zoledronat	10	9.61
	Intravenous	9	90.00
	Peroral	0	0.00
	No information	1	10.00
	Ibandronat	28	26.92
	Intravenous	23	82.14
	Peroral	3	10.71
	No information	2	7.14
	Alendronat	13	12.50
	Intravenous	1	7.69
	Peroral	10	76.92
	No information	2	15.38
	No information	6	5.76
Duration antiresorptive therapy	Years (mean ± SD)	4.08 ± 3.30	
Administration schedule antiresorptive Therapy	Times per Year		
	1	8	7.69
	2	36	34.61
	3	1	0.96
	4	21	20.19
	12	11	10.57
	52	8	7.69
	No information	19	18.26
Indication antiresorptive therapy	Osteoporosis	67	64.42
	Osseous metastasis	14	13.46
	Multiple myeloma	5	4.80
Systemic co-factors	Diabetes	5	4.80
	Anemia	2	1.92
	Anemia and diabetes	1	0.96
	Secondary osteoporosis	3	2.88
	Prevention SRE	1	0.96
	Immunomodulators	6	5.76
	Variables related to number of extractions		
Parameter	Category	Result	Percentage (%)
Extractions	Total	203	
Extraction site	Upper jaw	95	46.80
	Molar	43	45.26
	Premolar	31	32.63
	Front tooth	21	22.11
	Lower jaw	108	53.20
	Molar	52	48.15
	Premolar	34	31.48
	Front tooth	22	20.37
Current/previous antiresorptive therapy at time of extraction	Current Previous	151 52	74.38 25.62
Surgical technique wound closure	Mucoperiosteal flap	190	93.59
	Xenogenic graft	4	1.97
	Mucosal flap	2	0.98
	No flap	3	1.47
	No information	4	1.97
Antibiotic	Amoxicillin	130	64.03
	Co-amoxicillin	35	17.24
	Clindamycin	27	13.30
	No informatio	11	5.41
Duration antibiotic	Weeks (mean ± SD)	2.20 ± 0.79	
Inflammatory complications	Yes	60	29.55
	Dehiscence and/or,	14	23.33
	With revision	9	64.28
	No ARONJ	4	44.44
	ARONJ	5	55.56
	Without revision	5	35.71
	No ARONJ	3	60.00
	ARONJ	2	40.00
	Pain and/or,	27	45.00
	Redness and/or,	11	18.33
	Hematoma and/or,	21	35.00
	Swelling	10	16.67
	No	142	69.95
	No information	1	0.49

**Table 3:** Number of extraction sites with ARONJ depending on localization (same/different than extraction site) and timing of ARONJ occurrence (before extraction/after extraction).

Localization	Before Extraction	After Extraction	Before and After
Same	8	3	3
Different	9	3	0
Same and Different	1	1	1

**Table 4:** Characteristics of ARONJ patients: 21 patients total, (*) = characteristics of the 4 patients with ARONJ after extraction at same location without previous ARONJ.

	Variables Related to Number of Patients		
Parameter	Category	Result	Percentage
Age	Years (mean ± SD ^1^)	73.48 ± 13.50 (56.00 ± 7.41 *)	
Gender	Male	4 (2 *)	19.05 (50.00 *)
	Female	17 (2 *)	80.95 (50.00 *)
Smoking	Yes	7 (3 *)	33.33 (75.00 *)
	No	14 (1 *)	66.67 (25.00 *)
Risk profile	Low	12 (1 *)	57.14 (25.00 *)
	Medium	3 (1 *)	14.29 (25.00 *)
	High	6 (2 *)	28.57 (50.00 *)
Antiresorptive drug/ Route of administration	Prolia^®^ (subcutaneous)	7 (1 *)	33.33 (25.00 *)
	Xgeva^®^ (subcutaneous)	4 (2 *)	19.04 (50.00 *)
	Zoledronat	2 (0 *)	9.52 (0.00 *)
	Intravenous	1 (0 *)	50.00 (0.00 *)
	Peroral	0 (0 *)	00.00 (0.00 *)
	No information ^2^	1 (0 *)	50.00 (0.00 *)
	Ibandronat	4 (0 *)	19.04 (0.00 *)
	Intravenous	4 (0 *)	100.00 (0.00 *)
	Peroral	0 (0 *)	0.00 (0.00 *)
	No information	0 (0 *)	0.00 (0.00 *)
	Alendronat	4 (1 *)	19.04 (25.00 *)
	Intravenous	0 (0 *)	00.00 (0.00 *)
	Peroral	3 (1 *)	75.00 (100.00 *)
	No information	1 (0 *)	25.00 (0.00 *)
Duration antiresorptive therapy	Years (mean ± SD)	4.85 ± 4.93 (2.00 ± 0.81 *)	
Indication antiresorptive therapy	Osteoporosis	12 (1 *)	57.14 (25.00 *)
	Osseous metastasis	4 (2 *)	19.04 (50.00 *)
	Multiple myeloma	2 (0 *)	9.52 (0.00 *)
Systemic co-factors	Diabetes	1 (0 *)	4.76 (0.00 *)
	Anemia	1 (0 *)	4.76 (0.00 *)
	Anemia and diabetes	0 (0 *)	0.00 (0.00 *)
	Secondary osteoporosis	0 (0 *)	0.00 (0.00 *)
	Prevention SRE ^3^	0 (0 *)	0.00 (0.00 *)
	Immunomodulators	1 (1 *)	4.76 (25.00 *)
	Variables related to number of extractions		
Parameter	Category	Result	Percentage (%)
Extractions	Total	29 (4 *)	37.93 (75.00 *)
Extraction site	Upper jaw	11 (3 *)	27.27 (66.67 *)
	Molar	3 (2 *)	45.45 (33.33 *)
	Premolar	5 (1 *)	27.27 (0.00 *)
	Front tooth	3 (0 *)	62.07 (25.00 *)
	Lower jaw	18 (1 *)	33.33 (100.00 *)
	Molar	6 (1 *)	50.00 (0.00 *)
	Premolar	9 (0 *)	16.67 (0.00 *)
	Front tooth	3 (0 *)	37.93 (75.00 *)
Current/previous antiresorptive therapy at time of extraction	Current Previous	19 (4 *) 10 (0 *)	65.52 (100.00 *) 34.48 (0.00 *)
Inflammatory complications	Yes Dehiscence and/or, Pain and/or, Redness and/or, Hematoma and/or, Swelling No	9 (4 *) 7 (4 *) 6 (3 *) 3 (1 *) 0 (0 *) 0 (0 *) 20 (0 *)	31.03 (100.00 *) 77.78 (100.00 *) 66.67 (75.00 *) 33.33 (25.00 *) 0.00 (0.00 *) 0.00 (0.00 *) 68.97 (0.00 *)

(*) = characteristics of the 4 patients with ARONJ after extraction at the same location without previous ARONJ ; ^1^ SD = Standard deviation; ^2^ no information found in medical record; ^3^ SRE = skeletal related events

## Data Availability

The data presented in this study are available on request from the corresponding author. The data are not publicly available due to privacy restrictions.
